# Association between blood glucose levels and Glasgow Outcome Score in patients with traumatic brain injury: secondary analysis of a randomized trial

**DOI:** 10.1186/s13063-022-06005-5

**Published:** 2022-01-15

**Authors:** Tao Yuan, Hongyu He, Yuepeng Liu, Jianwei Wang, Xin Kang, Guanghui Fu, Fangfang Xie, Aimin Li, Jun Chen, Wenxue Wang

**Affiliations:** 1 Department of Neurosurgery, The Affiliated Lianyungang Oriental Hospital of Xuzhou Medical University, Lianyungang, Jiangsu, 222042 China; 2 Centre for Clinical Research and Translational Medicine, The Affiliated Lianyungang Oriental Hospital of Xuzhou Medical University, Jiangsu Province, 222042 China; 3 Department of Neurosurgery, Lianyungang No.1 People’s Hospital, Lianyungang, Jiangsu Province, 222042 China

**Keywords:** Traumatic brain injury, Glasgow Outcome Score, Blood glucose, Hyperglycemia, Insulin therapy

## Abstract

**Background:**

Blood glucose levels that are too high or too low after traumatic brain injury (TBI) negatively affect patient prognosis. This study aimed to demonstrate the relationship between blood glucose levels and the Glasgow Outcome Score (GOS) in TBI patients.

**Methods:**

This study was based on a randomized, dual-center, open-label clinical trial. A total of 208 patients who participated in the randomized controlled trial were followed up for 5 years. Information on the disease, laboratory examination, insulin therapy, and surgery for patients with TBI was collected as candidate variables according to clinical importance. Additionally, data on 5-year and 6-month GOS were collected as primary and secondary outcomes, respectively. For multivariate analysis, a generalized additive model (GAM) was used to investigate relationships between blood glucose levels and GOS. The results are presented as odds ratios (ORs) with 95% confidence intervals (95% CIs). We further applied a two- piecewise linear regression model to examine the threshold effect of blood glucose level and GOS.

**Results:**

A total of 182 patients were included in the final analysis. Multivariate GAM analysis revealed that a bell-shaped relationship existed between average blood glucose level and 5-year GOS score or 6-month GOS score. The inflection points of the average blood glucose level were 8.81 (95% CI: 7.43–9.48) mmol/L considering 5-year GOS as the outcome and were 8.88 (95% CI 7.43−9.74) mmol/L considering 6-month GOS score as the outcome. The same analysis revealed that there was also a bell relationship between average blood glucose levels and the favorable outcome group (GOS score ≥ 4) at 5 years or 6 months.

**Conclusion:**

In a population of patients with traumatic brain injury, blood glucose levels were associated with the GOS. There was also a threshold effect between blood glucose levels and the GOS. A blood glucose level that is either too high or too low conveys a poor prognosis.

**Trial registration:**

ClinicalTrials.gov NCT02161055. Registered on 11 June 2014.

**Supplementary Information:**

The online version contains supplementary material available at 10.1186/s13063-022-06005-5.

## Introduction

Traumatic brain injury (TBI) leads to death and disability in patients. Secondary brain injury significantly affects the prognosis of patients with TBI, while hyperglycemia is one of the important factors inducing secondary brain injury [1]. Recent studies have confirmed that hyperglycemia aggravates nerve damage [2]. Acute hyperglycemia following TBI, which is defined as a blood glucose level greater than 200 mg/dL (11 mmol/L) during the early phase of injury, is a common symptom in patients with severe TBI [3, 4]. Acute hyperglycemia following TBI is thought to be a physiological reaction that is essential for supporting the high metabolism in the brain [3, 5]. However, acute hyperglycemia was also reported to adversely affect outcomes of patients with TBI [4, 6] by exacerbating the secondary injury [7]. Since maximum clinical observations agreed on the existence of a close relationship between acute hyperglycemia and poor outcomes in patients with TBI [4, 6–10], early intervention for acute hyperglycemia in response to TBI is clinically recommended [11]. However, in clinical practice, hypoglycemia, which results from excess blood glucose control, was also found to adversely affect the TBI patient outcomes [12].

Blood glucose levels that are either too high or too low after TBI negatively affect the patient prognosis [13, 14]. Therefore, the control of the blood glucose levels in these patients has become an urgent problem due to its direct relationship with prognosis. It has been hypothesized that adequate control of acute hyperglycemia is necessary for patient benefits [11, 12]. A previous randomized controlled trial (RCT) confirmed this hypothesis [15], and 7–13 mmol/L is recommended as the target blood glucose range in insulin therapy. This study attempted to confirm our previous findings with advanced data-analysis tools to supply more information on the target blood glucose range to guide clinical practice.

## Methods

### Patients

This RCT was registered at ClinicalTrials.gov (NCT02161055) on June 5, 2014, before patient recruitment, which started in June 2014 and ended in December 2016. This was a randomized, parallel-assignment, open-label, controlled clinical trial conducted at the Affiliated Lianyungang Oriental Hospital of Xuzhou Medical University and Lianyungang No.1 People’s Hospital, China. This protocol followed the Declaration of Helsinki and was approved by the Lianyungang Oriental Hospital Medical Ethics Committee and the Medical Ethics Committee of Lianyungang No.1 People’s Hospital, China. According to the Standards of Medical Care in Diabetes [16], hyperglycemia was defined as a rapid increase in blood glucose > 7 mmol/L (126 mg/dL). Eligible patients with hyperglycemia after severe TBI were randomly assigned to either the intensive insulin therapy (IIT) group or the non-IIT group at a ratio of 3:1. The IIT group was further subdivided into three subgroups based on the target blood glucose level: 4.4–7.0 mmol/L (strict-control group), 7.1–10.0 mmol/L (moderate-control group), and 10.1–13.0 mmol/L (slight-control group). Computerized randomization was accomplished by investigators who could not contact the participants directly. Patients, outcome assessors, and statisticians were blinded to the information regarding grouping. If eligible patients consented to the trial, sealed opaque envelopes with a randomly assigned serial number containing the accepted treatment programs were opened. The patients then underwent the corresponding treatment measures. In the case of any error or disclosure regarding randomization, a new randomization sequence was generated starting from the problematic serial number and applied to subsequent patients.

### Inclusion and exclusion criteria

The inclusion criteria were as follows: (1) clinical diagnosis of severe closed TBI [17], (2) computed tomography (CT) confirmation of severe closed TBI, (3) severe TBI following the indications for craniotomy, (4) blood glucose levels of > 7.0 mmol/L measured twice by rapid examination within 2 h after admission, (5) Glasgow Coma Score (GCS) of 3–8, (6) age 18–80 years, and (7) no history of diabetes mellitus. The exclusion criteria were as follows: (1) patients with multiple physical injuries, (2) patients with diabetic nephropathy with hemodialysis dependence, (3) patients with neurological disorders before craniocerebral trauma, (4) patients with a history of diabetes before craniocerebral trauma, (5) bilaterally dilated pupils, and (6) refusal by the patient’s next of kin. Moreover, patients were withdrawn from the trial under the following conditions: (1) discontinuation of the trial was necessary from a medical point of view,or (2) a request was received from the patient’s family to stop the trial.

### Blood glucose control in insulin therapy

Within the first week of hospitalization, rapid blood glucose levels were recorded once every 2 h in each group. Blood glucose measurement was performed by collecting capillary blood obtained from the tip of the ring finger to determine blood glucose levels. Blood was collected from the same finger to ensure consistent measurement. To measure the blood glucose level of a patient undergoing transfusion, blood was collected from the tip of the finger of the limb without transfusion to ensure the accuracy of measurement.

In the IIT group, blood glucose levels were monitored and controlled according to the Yale Insulin Infusion Protocol [18]. According to the Yale Insulin Infusion Protocol, the amount of insulin (U) = [fasting blood glucose (mmol/L) × 18–100] × 10 × body weight (kg) × 0.6/(1000 × 2). Insulin for injection (400 U/10 ml) was obtained from Wan-bang Biochemical Pharmaceutical Co., Ltd., China (Lot # 1307230, 1302225, and 1307210). Insulin was infused into the vein at a rate of 0.1 U/(kg · h) using a micropump. During this period, blood glucose levels were monitored once every 2 h, and insulin doses were adjusted accordingly. If blood glucose levels were higher than the target value, the insulin dose was gradually increased by 1–2 U/h. When the blood glucose levels reached the target value, the insulin dose was gradually decreased until its administration was terminated.

In contrast, in the non-IIT group, rapid blood glucose level measurement was performed once every 2 h. When blood glucose levels were ≤ 13.0 mmol/L, no intervention was performed. In the cases with a blood glucose level > 13.0 mmol/L, insulin was subcutaneously injected regularly. Insulin was administered once every 8 h in a fasting state, whereas during venous or enteral nutrition infusion, it was infused 30 min before the nutrition infusion. When the blood glucose level reached ≤ 13.0 mmol/L, insulin infusion was terminated.

### Measurement of dependent variables

During the original RCT, patients were treated using the following uniform protocol. (1) According to the Guidelines for the management of severe TBI [19], craniotomy for TBI was performed primarily to decompress and remove hematomas. (2) During the procedure, all patients underwent ventricular puncture. Cerebrospinal fluid (CSF) was collected for biochemical analysis and cell culture. (3) All patients were closely monitored in the intensive care unit (ICU) of the Department of Neurosurgery. (4) Therapeutic protocols for severe TBI were used. (5) Glucocorticoids, which can cause disorders in glucose metabolism, were not regularly applied to either group. (6) During intravenous injection, glucose and insulin were mixed in a ratio of 5 g (glucose) to 1 U (insulin) to minimize the effects of exogenous glucose on blood glucose levels. When the patients finished the RCT, routine rehabilitation and daily life training were organized. The blood glucose, serum insulin, and blood glycosylated hemoglobin levels, as well as the CSF levels of glucose, lactic acid, and chloride, were monitored. CSF was collected during surgery and obtained 1 week after surgery by lumbar puncture. GCS was used to evaluate the severity of the patient's condition, and a recording sheet was used to assess the patient’s condition at hospital admission. The Acute Physiology and Chronic Health Evaluation II (APCHE II) score was recorded on each subsequent day in the ICU.

### Outcome measurement

The outcome of this study was the 5-year Glasgow Outcome Score (GOS). In supplementary analyses, we also analyzed the 6-month GOS. GOS definitions were as follows: 1, death; 2, persistent vegetative state; 3, severe disability (conscious but disabled), needing daily support; 4, moderate disability (disabled but independent); and 5, good recovery, normal active life with minimal deficits.

### Statistical analysis

Continuous variables that conformed to a normal distribution are described as the mean (SD), while those not conforming to a normal distribution are described as the median (Q1–Q3). Categorical data are presented as numbers and percentages. We selected these confounders based on the basis of their associations with the outcomes of interest or a change in effect estimate of more than 10%. Supplementary tables S2-S4 show the associations of each confounder with the outcomes of interest. Multivariate regression analysis was used to determine the independent relationship between blood glucose levels and outcomes. Because a nonlinear relationship was identified between the blood glucose level and 5-year GOS, a generalized additive model (GAM), which fits a nonlinear relationship, was used in multiple variable analysis to identify the relationships between blood glucose levels and GOS. The results are presented as ORs with 95% confidence intervals (95% CIs).

We further applied a two-piecewise linear regression model to examine the threshold effect of blood glucose levels and GOS. The turning point for blood glucose level was determined using exploratory analyses, which involved moving the trial turning point along with the predefined interval and picking the point that yielded the maximum model likelihood. We also conducted a log-likelihood ratio test comparing the one-line linear regression model to the two-piecewise linear model, as described in previous analyses [20, 21]. The two-sided alpha level was set at 0.05. All statistical analyses were performed using Empower Stats (www.empowerstats.com, X&Y solutions, Inc., MA, USA) and R software version 3.6.1 (http://www.r-project.org).

## Results

### Characteristics and patient outcomes

A total of 208 participants who participated in the RCT were enrolled in this cohort and followed up to December 2020. Of the total participants, 26 were lost to follow-up, leaving 182 in the final analysis (Supplementary Material Figure S1). For these 182 patients, the median age was 50 (34–59) years, the number of GCS tests before surgery, those with GCS of 3 or 4 were 51 (28.02%), those with GCS 5 or 6 were 83 (45.60%), those with GCS 7 or 8 were 48 (26.37%), the mean APCHE II score before surgery was 28.73 (2.40), the mean blood glucose level before surgery was 19.08 (2.25) mmol/L, the mean glycosylated hemoglobin level during surgery was 5.83 (0.96)%, the mean CSF glucose level during surgery was 5.26 (0.81) mmol/L, the mean CSF lactic acid level during surgery was 4.06 (0.67) mmol/L, and the average blood glucose level over 7 days of insulin therapy was 9.05 (2.68) mmol/L. Among all patients, 147 were men and 114 exhibited pupil changes (Table 1).

**Table 1:** Baseline characteristics of participants (*n* = 182)

Characteristics	
Age (years), median (Q1–Q3)	50.00 (34.00–59.00)
Sex, *n* (*%*)	
Male	147 (80.77%)
Female	35 (19.23%)
Pupil changes, *n* (*%*)	
No	68 (37.36%)
Yes	114 (62.64%)
GCS before surgery, *n* (*%*)	
3 or 4	51 (28.02%)
5 or 6	83 (45.60%)
7 or 8	48 (26.37%)
APCHE II score before surgery, mean (SD)	28.73 ± 2.40
Blood glucose before surgery (mmol/L), mean (SD)	19.08 ± 2.25
GHb before surgery, %, mean (SD)	5.83 ± 0.96
CSF glucose during surgery (mmol/L), mean (SD)	5.26 ± 0.81
CSF LA during surgery (mmol/L), mean (SD)	4.06 ± 0.67
Average blood glucose level (mmol/L), mean (SD)	9.05 ± 2.68

*GCS* Glasgow coma score, *APCHE II* Acute Physiology and Chronic Health Evaluation II, *GHb* glycosylated hemoglobin, *CSF* cerebrospinal fluid, *LA* lactic acid

Outcomes were analyzed using GOS after following up patients for a minimum of 5 years. Good recovery (GOS score 5) was achieved in 39 (21.43%) patients, moderate disability (GOS score 4) was achieved in 56 (30.77%) patients, severe disability (GOS score 3) was achieved in 30 (16.48%) patients, persistent vegetative state (GOS score 2) was achieved in 5 (2.75%) patients, and 52 (28.57%) patients (GOS score 1) died, in which unfavorable outcome or death (GOS score < 4) was observed in 87 (47.80%) patients, and favorable outcome (GOS score ≥ 4) was observed in 95 (52.20%) patients (Table 2).

**Table 2:** Outcome of participants (*n* = 182)

Outcome, ***n*** (***%***)	6 months	5 years
**Glasgow Outcome Score (GOS)**	182 (100%)	182 (100%)
1(dead)	33 (18.13%)	52 (28.57%)
2 (persistent vegetative state)	16 (8.79%)	5 (2.75%)
3 (severe disability)	53 (29.12%)	30 (16.48%)
4 (moderate disability)	43 (23.63%)	56 (30.77%)
5 (good recovery)	37 (20.33%)	39 (21.43%)
**Stratified by GOS**		
Unfavorable outcome or dead (GOS < 4)	102 (56.04%)	87 (47.80%)
Favorable outcome (GOS ≥ 4)	80 (43.96%)	95 (52.20%)

### Relationships between blood glucose levels and GOS

**Fig. 1:**
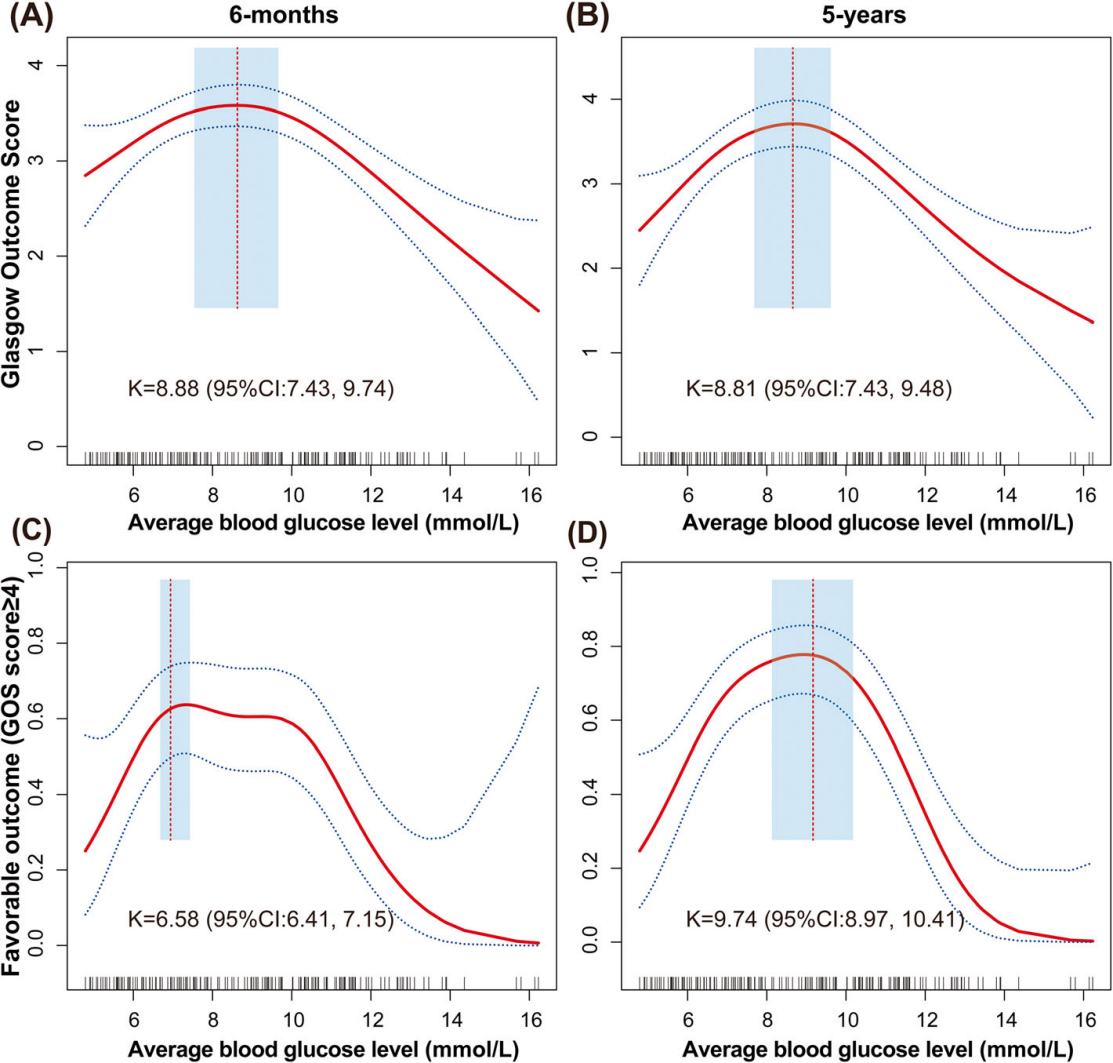
**A** A threshold, nonlinear association between the average blood glucose levels (mmol/L) and 6-month GOS was observed in a generalized additive model (GAM). The solid red line represents the smooth curve fit between variables. Blue bands represent the 95% confidence interval from the fit; 8.88 mmol/L (blood glucose level) were its inflection points (K). The 95% confidence interval of intervals of the infection points were 7.43 and 9.74. **B** A similar curve was found between the average blood glucose levels and 5-year GOS, in which 8.17 mmol/L was identified as the inflection point (K) with a 95% CI: 7.43–9.48. The same analysis revealed that there was also a bell relationship between average blood glucose levels and favorable outcome group (GOS ≥ 4) at 6 months **C** or 5 years **D**. The covariates were all adjusted for Glasgow coma score and Acute Physiology and Chronic Health Evaluation II score before surgery.

After adjustment for potential confounders, the one-line linear regression model revealed that the average blood glucose level has significance for the improvement of 5-year GOS (β: − 0.09, 95% CI: − 0.17 to − 0.01, *P* value: 0.0228) and 5-year favorable outcome (GOS score ≥ 4) (OR: 0.87, 95% CI: 0.77–0.98, *P* value: 0.0272) (Table 3). GAM was used for multivariate analysis; “Bell”-shaped relationships were observed between the average blood glucose levels in insulin therapy and 5-year GOS, in which 8.81 (95% CI: 7.43–9.48) mmol/L was identified as an inflection point (Fig. 1, Table 3). A similar curve was found between the average blood glucose levels and 6-month GOS, in which 8.88 (95% CI: 7.43–9.74) mmol/L was the inflection point (Fig. 1, Table 3). The same analysis revealed that there was also a bell relationship between average blood glucose levels and the favorable outcome group (GOS score ≥ 4) in 5 years or 6 months, (Fig. 1, Table 3). Compared with crude regression analyses, the associations did not change markedly after adjusting for GCS score and APCHE II score before surgery in the multivariate analyses (Supplementary tables S5-6).

**Table 3:** Threshold effect analysis of average blood glucose (mmol/L) with Glasgow coma score at 6 months and 5 years using piecewise linear regression

Outcome	6 months	5 years
**Glasgow coma score (GOS)**	***β*** **(95% CI)** ***P*** **value**	***β*** **(95% CI)** ***P*** **value**
Model I		
A linear inflection	− 0.09 (− 0.16, − 0.02) 0.0095	− 0.09 (− 0.17, − 0.01) 0.0228
Model II		
Inflection point (K)	8.88	8.81
< K	0.26 (0.09, 0.43) 0.0032	0.37 (0.18, 0.57) 0.0002
> K	− 0.34 (− 0.46, − 0.21) < 0.0001	− 0.40 (− 0.54, − 0.26) < 0.0001
Log likelihood ratio tests	< 0.001	< 0.001
95% confidence interval of Inflection point	7.43, 9.74	7.43, 9.48
**Favorable outcome (GOS ≥ 4)**	**OR (95% CI)** ***P*** **value**	**OR (95% CI)** ***P*** **value**
Model I		
A linear inflection	0.85 (0.75, 0.97) 0.0117	0.87 (0.77, 0.98) 0.0272
Model II		
Inflection point (K)	6.58	9.74
< K	5.44 (2.01, 14.72) 0.0008	1.68 (1.29, 2.19) 0.0001
> K	0.70 (0.59, 0.83) < 0.0001	0.30 (0.18, 0.48) < 0.0001
Log likelihood ratio tests	< 0.001	< 0.001
95% confidence interval of inflection point	6.41, 7.15	8.97, 10.41

*Abbreviations*: *CI* confidence interval, *OR* odds ratio, OR = exp(β). Adjusted for Glasgow coma score and Acute Physiology and Chronic Health Evaluation II before surgery

## Discussion

Insulin therapy is used to clinically control the dramatic increase in blood glucose levels following TBI and benefits TBI patient outcomes; in contrast, this therapy requires a target blood glucose range to direct the dose of insulin administered [22]. Unlike other brain pathologies, such as ischemic stroke and intracerebral hemorrhage, the target blood glucose range for TBI has not been well documented. Although 6.0–10.0 mmol/L was predicted as the appropriate target range of blood glucose [1, 22], this range has not been fully substantiated by clinical research. In a previous stud y[15], based on the effect of IIT on 3- and 6-month survival and GOS, we found that the medium-control IIT group exhibited the best GOS compared to the tight-control IIT group and slight-control IIT group, while mean glucose levels in the medium-control group fell in the range of 7.1 to 13.0 mmol/L. Therefore, we concluded that a blood glucose range of 7.1 to 13 mmol/L is a target range for IIT in TBI patients. In the present study, using prognostic data of 5-year GOS, we attempted to confirm previous findings and supply new information concerning the appropriate blood glucose range using advanced statistical tools.

During the multivariate GAM analysis of the relationship between insulin therapy groups and 5-year GOS, a “bell”-shaped relationship between the average blood glucose level at 7 days in IIT and 5-year GOS was observed, consistent with previous findings that a blood glucose level that is either too low or too high is harmful to patient outcomes [13, 14]. Furthermore, using curve-fitting and threshold effects analysis, we identified inflection points in the aforementioned curves. The inflection point of the average blood glucose level was 8.81 mmol/L considering 5-year GOS as the outcome. When using 6-month GOS as the outcome to identify the inflection points, similar values of 8.88 mmol/L were achieved. Combining our previous analytical results, we suggest that blood glucose levels during IIT should be controlled in the range of 7.1–13 mmol/L, and at the same time, levels should approach 8.81 mmol/L as closely as possible. This measurement would ensure that TBI patients benefit more from insulin therapy in terms of prognosis [23].

Due to the influence of the inclusion criteria, our study does have some limitations. We selected critical patients with a GCS of 3–8 and excluded patients with a GCS score of > 8. Additionally, patients with bilateral mydriasis were excluded due to their high mortality. We also excluded nonsurgical patients due to some interference differences between surgical and nonsurgical patients. The data used in this study were derived from an RCT that involved only a subset population of patients with TBI. Hence, our conclusion is suitable for a similar subset population. A cohort of patients with TBI of different severities may be a better choice to determine a target blood glucose range in future studies. In addition to the aforementioned choice bias, multicentre studies with larger sample sizes would help achieve a more general conclusion regarding the target glucose range in insulin therapy.

In conclusion, in a population of patients with traumatic brain injury, blood glucose levels were associated with the GOS. There was also a threshold effect between blood glucose levels and the GOS. A blood glucose level that is either too high or too low conveys a poor prognosis.

## Supplementary Information

**Additional file 1: Supplementary Material.** Association between blood glucose levels and Glasgow Outcome Score in patients with traumatic brain injury: Secondary analysis of a randomized trial.

## Data Availability

1. Supplementary data for this article can be found online at 10.5061/dryad.tx95x69xn 2. Additional file: Supplementary Material.pdf
