# Supplementary material for: Association between blood glucose levels and Glasgow Outcome Score in patients with traumatic brain injury: secondary analysis of a randomized trial
Source: Trials. 2022 Jan 15;23:38. doi: 10.1186/s13063-022-06005-5 (PMC8760745; doi:10.1186/s13063-022-06005-5)
Supplement: Supplementary file 1 — Additional file 1: Supplementary Material. Association between blood glucose levels and Glasgow Outcome Score in patients with traumatic brain injury: Secondary analysis of a randomized trial. [file 13063_2022_6005_MOESM1_ESM.pdf]

Supplementary Material

Association between blood glucose levels and Glasgow Outcome Score in patients with traumatic brain injury: Secondary analysis of a randomized trial

Tao Yuan[1†], Hongyu He[1†], Yuepeng Liu[2], Jianwei Wang[1], Xin Kang[1], Guanghui Fu[1], Fangfang Xie[1], Aimin Li[3], Jun Chen[3], *and* Wenxue Wang[1*]

*** Correspondence:** Wenxue Wang, 760020210075@xzhmu.edu.cn.

† Tao Yuan and Hongyu He contributed equally to this work.

[1] Department of Neurosurgery, the Affiliated Lianyungang Oriental Hospital of Xuzhou Medical University, 222042. Lianyungang, Jiangsu, China.

[2] Centre for Clinical Research and translational medicine, the Affiliated Lianyungang Oriental Hospital of Xuzhou Medical University, 222042, Jiangsu Province, China.

[3] Department of Neurosurgery, Lianyungang No.1 People's Hospital, Lianyungang, 222042, Jiangsu Province, China.

Contents

Figure S1: CONSORT 2010 Transparent Reporting of Trials: Flow diagram.

Table S1. Univariate analysis of GOS at 6 months or 5 years.

Table S2. Covariate examination and screening: VIF collinearity screening.

Table S3. The relationship between covariates and the 5-year GOS was examined one by one.

Table S4. Covariates were introduced into the basic model, and covariates were eliminated from the complete model to observe the change in the regression coefficient of the average blood glucose level (mmol/L).

Table S5. The relationship between average blood glucose level (mmol/L) and GOS and favourable outcome (GOS⩾4) at 6 months or 5 years.

Table S6. Threshold effect analysis of average blood glucose (mmol/L) with Glasgow coma score at 6 months and 5 years using piecewise linear regression

Figure S1: CONSORT 2010 Transparent Reporting of Trials: Flow diagram.

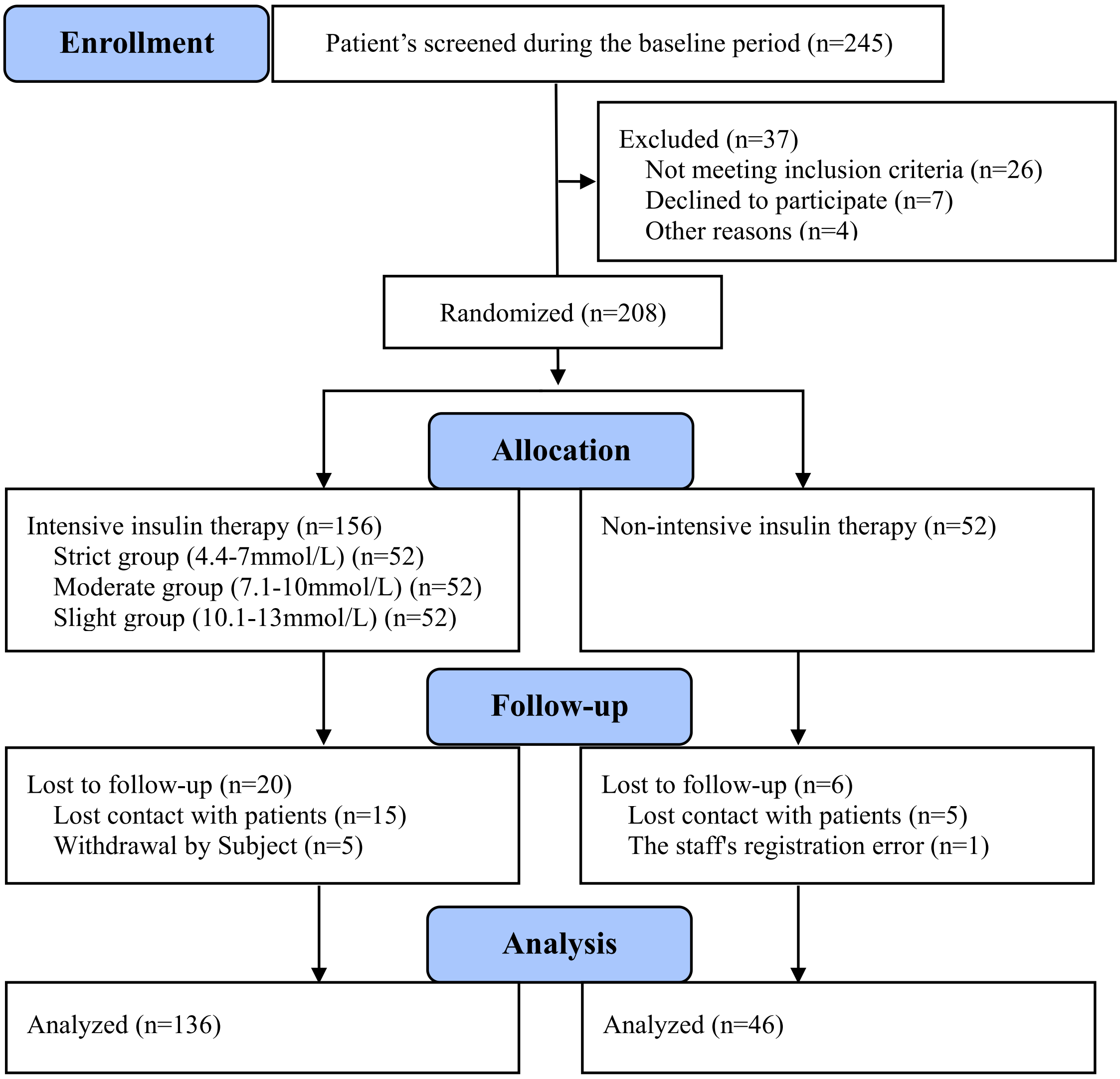

Table S1. Univariate analysis of GOS at 6 months or 5 years

	Statistics	6-month GOS	5-year GOS
		**β (95%CI) P value**	**β (95%CI) P value**
Sex, n (%)			
male	147 (80.77%)	Reference	Reference
female	35 (19.23%)	-0.17 (-0.67, 0.33) 0.5129	-0.13 (-0.70, 0.43) 0.6402
Age, year	46.73 ± 16.29	0.00 (-0.01, 0.01) 0.6641	-0.00 (-0.01, 0.01) 0.9376
Pupil changes, n(%)			
no	68 (37.36%)	Reference	Reference
yes	114 (62.64%)	-0.73 (-1.12, -0.33) 0.0004**	-0.72 (-1.17, -0.27) 0.0019**
GCS before surgery, n(%)			
3 or 4	51 (28.02%)	Reference	Reference
5 or 6	83 (45.60%)	0.67 (0.20, 1.13) 0.0052**	0.61 (0.09, 1.14) 0.0231*
7 or 8	48 (26.37%)	0.69 (0.17, 1.21) 0.0106*	0.65 (0.05, 1.24) 0.0342*
APCHE II score before surgery, mean±SD	28.73 ± 2.40	-0.18 (-0.25, -0.10) <0.0001**	-0.18 (-0.27, -0.09) 0.0001**
Blood GLU before surgery, mmol/L, mean±SD	19.08 ± 2.25	-0.13 (-0.22, -0.04) 0.0037**	-0.11 (-0.21, -0.01) 0.0256*
GHb before surgery, %, mean±SD	5.83 ± 0.96	-0.26 (-0.46, -0.05) 0.0142*	-0.20 (-0.43, 0.03) 0.0977
CSF GLU during surgery, mmol/L, mean±SD	5.26 ± 0.81	0.33 (0.09, 0.57) 0.0080*	0.26 (-0.02, 0.53) 0.0671
CSF LA during surgery, mmol/L, mean±SD	4.06 ± 0.67	-0.37 (-0.66, -0.08) 0.0141*	-0.28 (-0.61, 0.05) 0.0969
Average blood glucose level, mmol/L, mean±SD	9.05 ± 2.68	-0.09 (-0.16, -0.02) 0.0144*	-0.09 (-0.17, -0.01) 0.0334*

Abbreviations: CI, confidence interval. OR, odds ratio. GCS, Glasgow coma score. APCHE II, Acute Physiology and Chronic Health Evaluation II. BI, Barthel Index. GOS, Glasgow Outcome Score. GHb, Glycosylated haemoglobin. CSF, Cerebrospinal fluid. Glu, Glucose; LA, Lactic Acid. *P value<0.05, **P value<0.01.

Table S2. Covariate examination and screening: VIF collinearity screening

	Step 1	Step 2
Average blood glucose level (mmol/L)	1	1
Sex	1	1
Age (years)	1.1	1.1
Pupil changes	1.1	1.1
GCS before surgery	2.3	2.3
APCHE II score before surgery	3.4	3.3
Blood glucose before surgery (mmol/L)	2.1	2.1
GHb before surgery (%)	52037	2
CSF glucose during surgery (mmol/L)	1.1	1.1
CSF LA during surgery (mmol/L)	52080.6	NA*

Abbreviations: GCS, Glasgow coma score; APCHE II, Acute Physiology and Chronic Health Evaluation II; GHb, Glycosylated haemoglobin; CSF, Cerebrospinal fluid; LA, Lactic Acid. *The variable excluded by collinearity screening was CSF LA during surgery (mmol/L).

Table S3. The relationship between covariates and 5-year GOS one by one.

Covariates	N	term	beta	Se.	95%CI Low	95%CI High	P value
Sex	182	factor (Sex)2	-0.1347	0.2877	-0.6985	0.4291	0.6402
Age (years)	182	Age	-0.0005	0.007	-0.0142	0.0131	0.9376
Pupil changes	182	factor (Pupil changes)1	-0.7198	0.2283	-1.1672	-0.2724	0.0019**
GCS before surgery	182	factor (GCS)4	1.375	0.4136	0.5644	2.1856	0.0011**
		factor (GCS)5	1.4006	0.3825	0.651	2.1503	0.0003
		factor (GCS)6	1.2917	0.3741	0.5584	2.0249	0.0007
		factor (GCS)7	1.3083	0.4037	0.517	2.0996	0.0014
		factor (GCS)8	1.4861	0.4597	0.5852	2.387	0.0015
APCHE II score before surgery	182	APCHE II score	-0.178	0.0456	-0.2673	-0.0887	0.0001**
Blood glucose before surgery (mmol/L)	182	Blood glucose	-0.1124	0.0499	-0.2102	-0.0145	0.0256*
GHb before surgery (%)	182	GHb	-0.1963	0.1179	-0.4273	0.0348	0.0977
CSF glucose during surgery (mmol/L)	182	CSF glucose	0.2553	0.1386	-0.0163	0.527	0.0671

Abbreviations: GCS, Glasgow coma score; APCHE II, Acute Physiology and Chronic Health Evaluation II; GHb, Glycosylated haemoglobin; CSF, Cerebrospinal fluid. *P value<0.05, **P value<0.01.

Table S4. Introduction of covariates into the basic model and elimination of covariates from the complete model to determine changes in the regression coefficient of average blood glucose level (mmol/L).

Covariant	+/- term	Basic model<br>Average blood glucose level (mmol/L)	Complete model<br>Average blood glucose level (mmol/L)	Selected
	Initial regression coefficient	-0.0898	-0.0949	
Sex	factor (Sex)	-0.0928	-0.0901	
Age (years)	Age	-0.0904	-0.0933	
Pupil changes	factor (Pupil changes)	-0.0828	-0.0990	
GCS before surgery	factor (GCS)	-0.0959	-0.0788*	Yes
APCHE II score before surgery	APCHE II score	-0.0783	-0.0966*	Yes
GHb before surgery (%)	GHb	-0.0875	-0.0953	
CSF glucose during surgery (mmol/L)	CSF glucose	-0.0893	-0.0956	
CSF LA during surgery (mmol/L)	CSF LA	-0.0883	-0.0951	

* The change in the initial regression coefficient was more than 10%.

Table S5. The relationship between average blood glucose level (mmol/L) and GOS and favourable outcome (GOS⩾4) at 6 months or 5 years.

Exposure	Non-adjusted	Adjusted model
	β (95%CI) P value	β (95%CI) P value
**6-month GOS**	-0.09 (-0.16, -0.02) 0.0144	-0.09 (-0.16, -0.02) 0.0095
**6-month favourable outcome (GOS≥4)**	-0.14 (-0.25, -0.02) 0.0193	-0.16 (-0.28, -0.04) 0.0117
**5-year GOS**	-0.09 (-0.17, -0.01) 0.0334	-0.09 (-0.17, -0.01) 0.0228
**5-year favourable outcome (GOS≥4)**	-0.11 (-0.22, -0.00) 0.0491	-0.14 (-0.26, -0.02) 0.0272

Abbreviations: CI, confidence interval. GOS, Glasgow Outcome Score. Adjusted model adjusted for: Glasgow coma score and Acute Physiology and Chronic Health Evaluation II before surgery.

Table S6. Threshold effect analysis of average blood glucose (mmol/L) with Glasgow coma score at 6 months and 5 years using piecewise linear regression

Glasgow coma score (GOS)	6 months	5 years
	β (95%CI) P value	β (95%CI) P value
**Non-adjusted**		
Model I		
A linear Inflection	-0.09 (-0.16, -0.02) 0.0144	-0.09 (-0.17, -0.01) 0.0334
Model II		
Inflection point (K)	8.88	8.81
< K	0.32 (0.15, 0.49) 0.0003	0.44 (0.25, 0.64) <0.0001
>K	-0.38 (-0.51, -0.25) <0.0001	-0.44 (-0.58, -0.30) <0.0001
Log Likelihood Ratio Tests	<0.001	<0.001
95% confidence interval of Inflection point	7.43, 9.74	7.43, 9.42
**Adjust**		
Model I		
A linear Inflection	-0.09 (-0.16, -0.02) 0.0095	-0.09 (-0.17, -0.01) 0.0228
Model II		
Inflection point (K)	8.88	8.81
< K	0.26 (0.09, 0.43) 0.0032	0.37 (0.18, 0.57) 0.0002
>K	-0.34 (-0.46, -0.21) <0.0001	-0.40 (-0.54, -0.26) <0.0001
Log Likelihood Ratio Tests	<0.001	<0.001
95% confidence interval of Inflection point	7.43, 9.74	7.43, 9.48

Abbreviations: CI, confidence interval. OR, odds ratio, OR=exp(β). Adjusted for Glasgow coma score and Acute Physiology and Chronic Health Evaluation II before surgery.
